# Cannabinoids and Neurogenesis: The Promised Solution for Neurodegeneration?

**DOI:** 10.3390/molecules26206313

**Published:** 2021-10-19

**Authors:** Andrea Valeri, Emanuela Mazzon

**Affiliations:** IRCCS Centro Neurolesi “Bonino-Pulejo”, Via Provinciale Palermo, Contrada Casazza, 98124 Messina, Italy; andrea.valeri@irccsme.it

**Keywords:** cannabinoid receptors, neurogenesis, neuroregeneration, phytocannabinoids, synthetic cannabinoids

## Abstract

The concept of neurons as irreplaceable cells does not hold true today. Experiments and evidence of neurogenesis, also, in the adult brain give hope that some compounds or drugs can enhance this process, helping to reverse the outcomes of diseases or traumas that once were thought to be everlasting. Cannabinoids, both from natural and artificial origins, already proved to have several beneficial effects (e.g., anti-inflammatory, anti-oxidants and analgesic action), but also capacity to increase neuronal population, by replacing the cells that were lost and/or regenerate a damaged nerve cell. Neurogenesis is a process which is not highly represented in literature as neuroprotection, though it is as important as prevention of nervous system damage, because it can represent a possible solution when neuronal death is already present, such as in neurodegenerative diseases. The aim of this review is to resume the experimental evidence of phyto- and synthetic cannabinoids effects on neurogenesis, both in vitro and in vivo, in order to elucidate if they possess also neurogenetic and neurorepairing properties.

## 1. Introduction

Neurodegeneration is primarily defined as the progressive loss of neurons, though not limited to, in the brain. [1]. Neurodegeneration is the main hallmark and the common feature between the major neurodegenerative diseases, including Alzheimer’s disease (AD), Parkinson’s disease (PD) and amyotrophic lateral sclerosis (ALS), where resulting dementia and/or motor impairment lead inevitably to the loss of individual independence. The treatments proposed for these devastating diseases can fight one aspect of the many different causes: i.e., Aducanumab, a monoclonal antibody recently approved for AD treatment, focuses its action on reducing beta-amyloid (Aβ) accumulation, with decreased tau phosphorylation only in a small portion of patients. Moreover, as all drugs it comes with side effects, which can be headache or dizziness, but also temporary brain swelling and bleeding were reported [2]. AD, PD and ALS are not the only diseases that can lead to loss of neurons: vascular diseases such as ischemia and hemorrhagic stroke can occur in different brain areas, as well as infections or accidents [3,4,5]. For a long time, neurons were considered irreplaceable, so when trauma or injury succeeded in damaging an area of the brain, it was believed its function was lost forever. However, some observations and hypothesis theorized the presence of immature neurons or undifferentiated cells in the brain that can differentiate in neurons, and several tests proved their existence: in one of the oldest experiments, in 1965, the autoradiography of rat brains, after injection of H^3^-thymidine, pointing at the dentate gyrus (DG) of the hippocampus as the site of neurogenesis. Interestingly, this process, also, seemed to continue, even if to a lesser extent, in adult rats [6].

*Cannabis sativa* plants contain different chemical species and more than 60 belong to the group of terpenophenolic compounds of cannabinoids [7]. The beneficial properties of *C. Sativa* are not a mystery, since historians found evidence of the use of this plant several centuries before Christ as painkiller or in shamanic rituals [8]. Cannabinoids that can be extracted directly from the plant fall under the name of phytocannabinoids, where Δ^9^-tetrahydrocannabinol (THC) and cannabidiol (CBD) are the major and most famous: THC is known for being psychoactive, while CBD does not possess any psychotropic effect and therefore, it represents a better candidate for medical use [9]. It is possible to extract cannabinoids from the plant using different methods: the organic solvent method is the first of the conventional ones, but the use of heat and acids may provoke modifications in the desired compound; the supercritical fluid extraction often use the CO_2_ and a co-solvent, in order to overcome the inability of CO_2_ to dissolve polar compounds. The supercritical fluid extraction is also reported as method to obtain a high yield of non-psychoactive compounds for C. Sativa; the other methods are dynamic maceration, ultrasound-assisted extraction and microwave-assisted extraction [10].

The potential applications of CBD, and cannabinoids more in general, in the treatment of several diseases, think anti-inflammatory [11], immunomodulatory [12] and neuroprotective properties [13], lead to the synthesis of artificial cannabinoids compounds, named synthetic cannabinoids. To date, Food and Drug Administration (FDA) approved four different drugs based on cannabinoids and two of them contain synthetic cannabinoids: Cesamed or Nabilone, whose formula recalls THC one [14] and Marinol or Dronabinol [15], a synthetic THC. The other two drugs are Epidiolex, also approved in Europe, based on plant-extracted CBD [16] and Sativex, composed of THC and CBD in a proportion 1:1 [17]. Since 1945 cannabinoids gain more and more attention, but only after the beginning of the new millennia the studies and PubMed publications regarding cannabinoids highly increased, from 379 in 2000 to 2473 in 2020. Cannabinoids and neurogenesis produced 232 results in PubMed which increase year by year, so the attention of the scientific community is slowly attracted by this almost unexplored association. To date, there is not a specific drug selected for boosting neurogenesis. Previous works focus their attention on the neuroprotective properties of cannabinoids. More in general, the prevention of neurodegenerative diseases gained a lot of attention from the scientific community, probably due to lack of drugs capable to resolve the consequences of the disease. However, when the damage is already there, in case of a non-predictable trauma or in the neurodegenerative diseases, the need of a solution cannot be ignored. Neurogenesis is a process not so deeply investigated, indeed to our knowledge the association between cannabinoids and neurogenesis was not reviewed before. The aim of this review is to focus the attention on the phyto- and synthetic cannabinoids effects on neurogenesis, and with the aid of in vitro and in vivo studies we can explore their beneficial effects in a process not so highly represented in literature, but very important for therapeutic point of view.

## 2. Methodology

**Figure 1:**
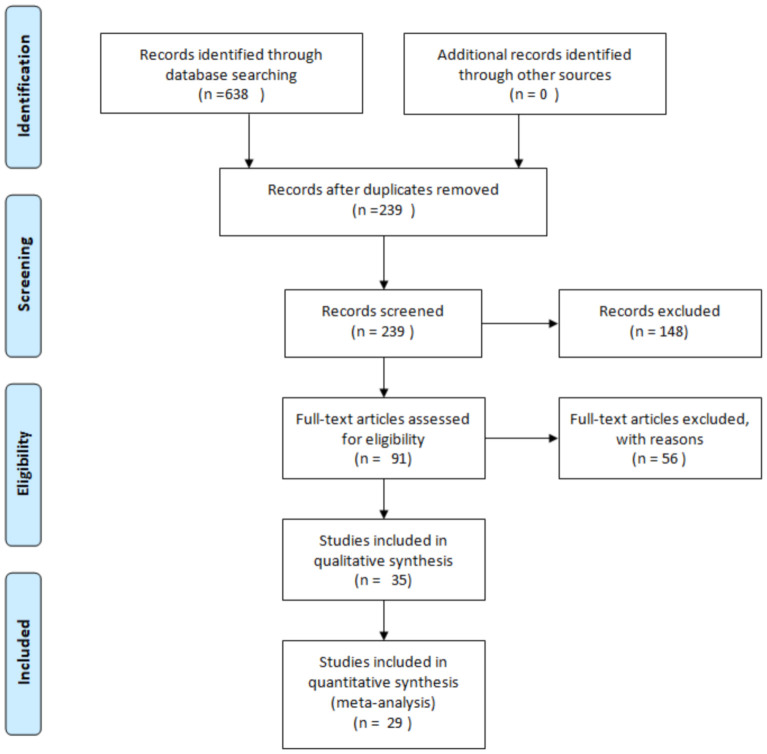
Prisma flow diagram of the methodology for selecting the review articles.

The selected publications range from 2005 to 2021. To retrieve he evidence, we used the PubMed database, with the keywords “cannabinoids”, “neurogenesis”, “cannabis”, “neuronal differentiation”, “neuroregeneration”, “Alzheimer’s Disease”, “Parkinson’s Disease”, “Huntington’s Disease”, “dementia”, “stroke”, “ischemia” and “stress”. The articles selected were the ones that describe in vitro and in vivo experiments and showing how the effects cannabinoids, both natural and synthetic, can influence neurogenesis. Reviews and articles not relevant for our topic and not in English were excluded from our analyses, as the Prisma flow diagram reports in Figure 1 [18].

## 3. Neurogenesis

Adult neurogenesis occurs thanks to a sub-population of stem cells, called neural stem cells (NSCs). They have self-renew capacities and can differentiate into brain cells, such as neurons, oligodendrocytes and astrocytes. Two main areas were identified as niches of neurogenesis in the adult brain: one is the subventricular zone of the lateral ventricle (SVZ) and the second is the subgranular zone of the hippocampus (SGZ) [19].

Different methods have been used to evaluate the existence and the extent of neurogenesis in adults. To evaluate if cells efficiently replicate, substances such as H^3^-thymidine or bromodeoxyuridine (BrdU) can be used. The majority of the experiments reported in this review will use BrdU as an evaluator of cell replication. Incorporation of BrdU can be observed using immunohistochemistry for phenotypical analysis and cells quantification. It has to be remembered, however, that the incorporation of BrdU or nucleotides analogs is considered as indicator of DNA synthesis, so other methods should exclude other ongoing processes, such as DNA reparation. Another method involves retroviruses because some of them do not possess the nuclear importation mechanism, so their genome can be integrated into the host genome only during mitosis. Transgene expression in the virus genome, such as a fluorescent protein, provides a good indicator of cell replication. However, this method may be invasive, since it requires a direct injection into the brain using stereotaxic surgery. Luckily, the maturation process of the neurons can be tracked following different markers expression. In the works reported in this review, the authors used mainly doublecortin (DCX), a protein associated to the microtubules and found expressed in neuronal progenitors. Other markers are calretinin (CR), whose expression differentiates post-mitotic immature neurons from intermediate neuronal progenitors. Antigen Ki-67 (Ki-67) is a marker of cell proliferation. β-tubulin isoform III (Tuj1) is a protein associated with microtubule, expressed during neuronal development [20].

The accepted model of hippocampal neurogenesis divides the process into six steps, lasting 2–4 months from proliferation to full integration of the new cell into the neural circuit. Each step is characterized by the expression of a specific marker, which makes it possible to monitor the development process. At stage 1, the radial glia-like cells express glial fibrillary acidic protein (GFAP) and share with astrocytes some common features, such as electrophysiological properties. Nestin expression distinguishes these cells from astrocytes. When they actively divide, the daughter cells express nestin too, and are always negative for polysialylated-neural cell adhesion molecule (PSA-NCAM). Sex determining region Y-box 2 (SOX2) is very important to maintain self-renew properties of NSCs through the repression of neurogenic differentiation 1 (NeuroD1) and regulation of the nuclear receptor subfamily 2 group E member 1 (TLX). From stage 2 to 4, the cells are high proliferative and can be divided into two subgroups, depending on their expression of DCX, and they are no more GFAP positive. At this point, a small portion of cell population starts to show the sodium current, as an early marker of neuronal differentiation. A third subpopulation starts to develop, and they are named neuroblasts, positive for DCX but negative for nestin. During these stages, NeuroD1, previously repressed, regulates the differentiation along with prospero related homeobox gene (Prox1). At stage 5 the cells maintain the expression of DCX, but other markers are ready to be expressed: CR characterize the early post mitotic stage and hexaribonucleotide binding protein-3 (NeuN) expression gives another hint of the post-mitotic status of these new cells. These neurons are still immature and will undergo a selection process prior to integration into the neural network. At the last stage, the 6th, cells repress CR and begin to express calbindine, a protein that acts as a calcium collector and binder. At this point, the neurons find their place in the circuit [21,22].

## 4. Cannabinoid Receptors: CB1, CB2, TRPV1, GPR55 and PPARγ

There are different cannabinoid receptors in the human nervous system, and they will be treated one by one in the following sections.

### 4.1. Cannabinoid Receptor 1

Cannabinoid receptor 1 (CBR1 or CB1) was first cloned from rat cerebral cortex and belongs to the G protein-coupled family receptors. In the brain, it was mainly found in hippocampus and cerebral cortex, but also in basal ganglia and cerebellum. It is the main target of arachidonyol ethanolamide (AEA) and THC [23,24]. In humans, the gene *CNR1* code for the receptor and its crystal structure was elucidated, also during the binding to its agonists [25,26]. CB1 has seven transmembrane domains and two terminal tails, one N-terminal in the extracellular part and one intracellular C-terminal part, plus three extracellular and three intracellular loops. Crystal structure studies reveal a marked difference in the N-terminal part of CB1 compared to other members of G protein-coupled receptors: CB1 showed α-helices between the first and the second extracellular loops, while other G-coupled receptors crystal structures contain a disulfide-crosslinked extracellular two loop structure [27]. The subcellular localization of CB1 is the axon terminal or the pre-terminal zone, presynaptically, and this is important for the endocannabinoid signaling pathway. It has also been found that the majority of these axon terminals have gamma-aminobutyric acid (GABA)ergic properties [28], but CB1 is also present in glutamatergic neurons of cortex and hippocampus, as well as cholinergic, noradrenergic and serotoninergic terminals [29]. Noteworthy, it has been reported the presence of CB1, also intracellularly, in particular, associated to endosomal and lysosomal compartment and indeed AEA activation of CB1 release calcium in the cytoplasm [30]. CB1 located on the plasma membrane, on the other hand, reduces the amount of intracellular calcium content and the suppression of calcium flux will reduce neurotransmission [31]. The canonical signaling pathway of CB1 involves the recruitment of β-arrestin 1 and 2, which exert different effects: β-arrestin 1 promotes the activation of mitogen-activated protein kinases (MAPKs) and regulates genes expression, while β-arrestin 2 acts on receptor desensitization and signal termination. Moreover, the signal of CB1 acts on c-Jun N-terminal kinase (JNK) and phosphoinositide 3-kinases/protein kinase B (PI3K/AKT), this latter responsible for neurons survival [29]. Taken together, MAPKs, JNK and PI3K/AKT play an important role in inducing transcription factors such as brain-derived neurotrophic factor (BDNF), which acts in support of neuronal survival, and early growth response protein 1 (KROX-24), which is expressed during development of Schwann cells and is strictly correlate with proliferation [32]. In addition, PI3K/AKT is a protective pathway for oligodendrocytes progenitors during trophic support deprivation and this preservation occurs via CB1 activation [33]. There is also a non-canonical pathway of CB1 signaling following activation, especially after binding with synthetic cannabinoids: through the activation of Gαi/o, extracellular signal-regulated kinases (ERK) can be phosphorylated, as well as AKT which can be phosphorylated in a Gβγ-dependent manner [34]. Moreover, the non-canonical pathway correlates to an increase of intracellular calcium and this can have an effect, not only in neurons, but also in astrocytes: the endocannabinoid signal from pyramidal neurons acts on astrocytes CB1, inducing the increase of their internal amount of calcium and then a signal back to neurons, and this could be important in the remodeling of synapsis [29,35]. It has been shown also a role of CB1 in the differentiation of neural progenitor into astroglial cells: during the development of the cortex, ERK signaling is activated for neuronal differentiation, but CB1 and endocannabinoid system influence the generation of astroglial cells probably attenuating Rap-1/B-Raf signaling and subsequently ERK activation [36].

### 4.2. Cannabinoid Receptor 2

Cannabinoid receptor 2 (CBR2 or CB2) was identified as a receptor expressed on the macrophages, isolated from spleen [37]. Initially, it was defined as “peripheral cannabinoid receptor”, since its presence was localized only outside the central nervous system [38]. Recent methods of research revealed its presence also in the brain, even if it is less expressed than CB1. It shares some similarities with CB1, they both belong to the G protein-coupled family with seven transmembrane domains, along with an extracellular N-terminus, an intracellular C-terminus, three extracellular and three intracellular loops [39]. The gene encoding for CB2 is *CNR2* and is conserved between different mammal species [40]. The structural differences between CB1 and CB2 became evident during crystal structure determination of CB2: the extracellular portion of CB2 is notably different, especially in helices I and II, and the N-terminal part does not seem to play a role in ligand binding [41]. Neurons and microglia both express CB2, as is expected since CB2 was first identified in immune cells. Its location seems to be mainly postsynaptic [42]. CB2 was found expressed in pyramidal neurons and in the ventral tegmental area, where exert its function in inhibiting of dopaminergic neurons. CB2 expression is also present in prefrontal cortex and striatum [43]. Like CB1, also CB2 activation has an effect on PI3K/AKT/mechanistic target of rapamycin complex (mTORC) and JNK pathways, so it can promote neuronal [44] and oligodendrocytes survival [33]. Moreover, PI3K/AKT/mTORC pathway is responsible for neuronal progenitors proliferation that involves a cyclin-dependent kinase inhibitor 1B protein (p27Kip1) inhibition via serum and glucocorticoid-regulated kinase 1 (SGK1), in turn, induced by mTORC1; this is coherent with the expression of CB2 in undifferentiated progenitors, where the proliferation is crucial, and its downregulation during neuronal differentiation. Indeed, p27Kip1 promotes neuronal differentiation and its phosphorylation is associated with the balance between self-renewal (proliferation) and survival, correlated with protein associated with Lin-7 (Pals1)/mTORC1 activity. Mention must also be made of the presence of CB2 in microglia as a regulator of its activation and, consequently, of neuroinflammation, which is one of the responsible factors for neuronal loss in many neurodegenerative diseases [45].

### 4.3. Transient Receptor Potential Cation Channel Subfamily V Member 1

Transient receptor potential cation channel subfamily V member 1 (TRPV1) is also known as capsaicin receptor or vanilloid receptor 1. It is a nonselective cation channel and, for its ion channel nature, is expressed on plasma membrane. Like the other members of TRPs family, it shows six transmembrane segments and between fifth and the sixth the pore loop can be found. Studies on its crystal structure revealed high dynamicity of the outer pore domain and of the hydrophilic pocket defined by the external part of segments 3 and 4, and segments 4 to 6, while segments 1 to 4 were found to be stable and are used as an anchor where the other subunits move to bind to the ligands [46]. A large variety of stimuli can activate TRPV1, ranging from temperature to voltage, osmolarity and pH, but also some chemicals and, of course, cannabinoids can bind to the receptor and promote its activity [47]. Once stimulated, the opening allows the ions to enter into the cells, in particular, calcium ions, and after long stimulation, the channel goes to the desensitized state, where the channel is no longer able to respond to external stimuli [48]. TRPV1 is widely distributed, and, in particular, it can be found in the peripheral nervous system where it is localized in dorsal root ganglia, trigeminal ganglia and primary sensory neurons, with nociception function. It is not so highly express in the brain, but still, its presence can be detected in entorhinal cortex, olfactory bulb, hippocampus and hypothalamus [49]. In particular, in the hippocampus, it exerts a function as long-term potentiation (LTP) mediator of excitatory synapses, theorizing its function in synaptic plasticity also in other parts of the brain [50]. It is mainly located in the presynaptic region of the glutamatergic and GABAergic terminals, where it improves synaptic transmission through calcium influx inside neurons [51]. Knockout assay on cells and mice revealed the role of TRPV1 in neurogenesis, since TRPV1 is expressed in cultured neuronal progenitor cells (NPCs) and knocking out TRPV1 in NPCs promotes neurogenesis. Indeed, lack of TRPV1 increases cell proliferation in DG and SVZ. TRPV1 seems also expressed during the stages when proliferation needs to be regulated, becoming silent in the amplification stage, and then re-express in DCX positive neuronal progenitors. This is also confirmed by analyzing mice brains after exercise when neurogenesis was more prominent in TRPV1 knock-out mice [52].

### 4.4. G Protein-Coupled Receptor 55

G protein-coupled receptor 55 (GPR55) shares very little homologies with CB1 and CB2, respectively, 14% and 15%. It is a G-protein coupled receptor and its cloning is not correlated with cannabinoid research. Only years later, in silico analyses suggested its potential involvement in cannabinoid system [48,53]. GPR55 is part of the purine cluster of rhodopsin family, and it shares with CB1 and CB2 the seven transmembrane domains. GPR55 is able to couple with the G-proteins Gαq/12 and Gα13, which results in the activation of phospholipase C through the action of ras homolog gene family members A (RhoA) and Rho-associated protein kinase (ROCK). This results in different events, one is the increase of the intracellular levels of calcium, the second is the phosphorylation of ERK and the third is the phosphorylation of p38, which is part of the MAPKs signaling. GPR55 can, in such ways, regulate cyclooxygenase-2 (COX-2) so it plays a role also in inflammation regulation and microglia activation. Its downstream signaling also stimulates transcription factors such as nuclear factor kappa-light-chain-enhancer of activated B cells (NF-κB) [54,55]. It is expressed in a large variety of brain regions, such as the hippocampus, cerebellum and frontal cortex, but also in the immune system cell population, so it is found also in microglia. It can bind to AEA, 2-arachidonoyl glycerol (2-AG) and CBD, which exert antagonist function [48]. To assess the different properties of GPR55 receptor, studies on knockout animals were performed: mice lacking GPR55 seems to have impaired motor coordination and, also, a different heat perception, while there was no difference in anxiety-like behaviors, sensory-motor gating and fear-conditioning behavior. However, no immediate differences in neurodevelopment were noticed between knockout mice and wild type, suggesting that GPR55 may not play a very important role during development [56]. If this receptor is localized at a pre-synaptic or post-synaptic level is not fully elucidate [54]. Probably its localization is different between cells and tissue: GPR55 agonists seem to not have any effects on the post-synaptic store of calcium, while they exert their effects at a pre-synaptic level in hippocampal slices [57]. Its domain of influence is very different, from motor coordination to spatial and procedural memory, but it is also involved in pain and food intake. It is important to mention that GPR55 was found to be involved in alcohol consumption and aggression because its blockage leaded to aggressive and offensive responses in vivo [58].

### 4.5. Peroxisome Proliferator-Activated Receptor Gamma

Peroxisome proliferator- activated receptor gamma (PPARγ) belongs to the superfamily of nuclear receptors and its role, as well as the role of another type of PPARs, is to dimerize with retinoid X receptor-alpha after ligand interaction and translocate into the nucleus, where it regulates the expression of different genes. PPARγ is made by six domains, highly conserved between the different PPARs: two activation function domains, two zinc-fingers DNA binding domain, one hinge domain and one ligand-binding domain [59,60]. PPARγ is not a pure cannabinoid receptor, meaning that its activation provokes a large variety of events outside the endocannabinoid system: PPARγ controls indeed the expression of genes involved in the adipose tissue metabolism and generation, as well as inflammation and metabolic balance [61]. It has a role in the central nervous system, since is expressed also in neurons and NSCs, and among these functions, there are proliferation, development and differentiation of brain cells [62]. In particular, it has been shown how the expression of PPARγ peaks at 13.5 embryonic stage in the hindbrain of rats, indicating its role in development and, more in deep, in differentiation and apoptosis that occurs in that precise phase of rat embryonic development [63]. Regarding the adult rat brain, PPARγ is expressed in basal ganglia, in hippocampal formation and, also, in the hypothalamus [64].

## 5. Cannabinoids: Endocannabinoids, Phytocannabinoids and Synthetic Cannabinoids

### 5.1. Endocannabinoids: AEA and 2-AG

Endocannabinoids are endogenous compounds able to interact with cannabinoid receptors. There are different compounds, but the best characterized are anandamide (AEA), and 2-AG, both derivate from arachidonic acid.

AEA is the amide of arachidonic acid and ethanolamine and it was isolated from porcine brain during screening of cannabinoids receptor ligands. Its structure defined using mass spectrometry and nuclear magnetic resonance spectrometry [65]. Its synthesis used different pathways, while the principal involved calcium-dependent N-acyltransferase (NAT) in first phase, in order to create N-acylphosphatidylethanolamine (NAPE), that will be hydrolyzed by N-acylphosphatidylethanolamine phospholipase D (NAPE-PDL) to create N-acyl-ethanolamine (NAE) and phosphatidic acid [66]. NAE is often used as AEA synonym. Another pathway involved NAPE-phospholipase C (PLC) followed by phosphatase. A different route involves abhydrolase domain containing 4, N-Acyl phospholipase B (ABHD4) and the hydrolysis of acyl groups, followed by a second hydrolysis conducted by glycerophosphodiesterase 1 (GDE1). The last one requires the action of lyso-NAPE-PLD [67,68].

2-Arachidonoyl glycerol (2-AG) was isolated from canine intestine using methanol extraction, gas chromatography and nuclear magnetic resonance [69]. There are three identified pathways for the synthesis of 2-AG, while the first appears to be the main pathway in central nervous system: after the stimulation of receptors such as M1 or M3 muscarinic, phospholipase C-β (PLCβ) hydrolyzed an arachidonoyl-containing phosphatidylinositol 4,5-bisphosphate (PIP2), obtaining dyacilglycerol; then another hydrolysis occurs due to the action of diacylglycerol lipase (DAGL). There are two different DAGLs, named DAGLα and DAGLβ: the first one seems to be the main responsible for 2-AG synthesis in central nervous system, while the second regulates synaptic 2-AG and its synthesis during immune response [68,70].

### 5.2. Phytocannabinoids: THC, CBD and Effects on Neurogenesis

THC was isolated and its structure defined after chromatography of a hexane extract of hashish [71]. The synthesis of THC starts with the interaction between olivetolic acid and geranyl pyrophosphate (GPP), resulting in cannabigerolic acid (CBGA) after C-alkylation. Cannabigerolic acid, via Δ^1^-tetrahydrocannabinolic acid synthase, results in Δ^1^-tetrahydrocannabinolic acid (Δ^1^-THCA). A final step of decarboxylation, maybe due to temperature and light and not from enzymatic origin, transforms Δ^1^-THCA in THC [72].

Cannabidiol (CBD) shares with THC the synthesis pathway, since cannabigerolic acid is the common precursor of these two phytocompounds. CBGA is oxido-cyclized by cannabidiolic acid synthase instead of Δ^1^-THCA, resulting in cannabidiolic acid (CBDA) [68,73]. The decarboxylation, the same process described before, will transform CBDA in CBD. CBD was found to be able to interact with TRPV1 and lead to its desensitization [74].

**Figure 2:**
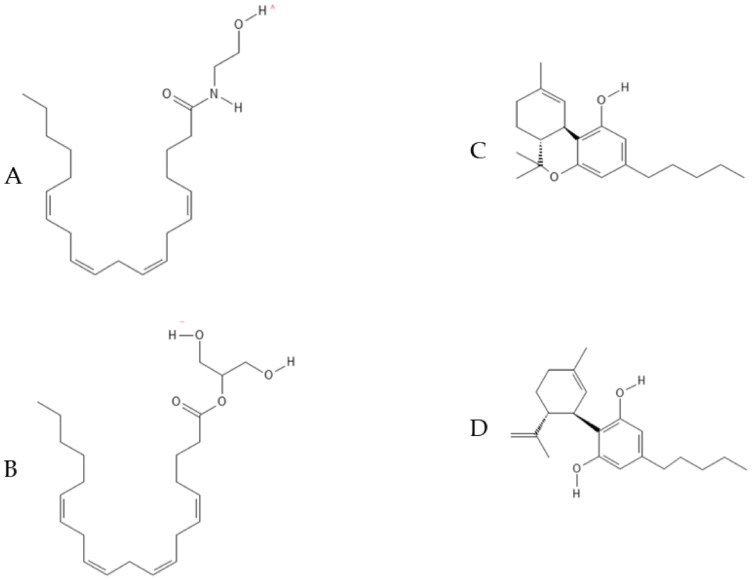
Chemical structure of the previously mentioned cannabinoids. (**A**) Anandamide (AEA); (**B**) 2-arachidonoyl glycerol (2-AG); (**C**) Δ^9^-tetrahydrocannabinol (THC); (**D**) cannabidiol (CBD).

The chemical structure of AEA, 2-AG, THC and CBD is reported in Figure 2.

### 5.3. Synthetic Cannabinoids: Classification, Nomenclature and Effects on Neurogenesis

Scientific research and the illegal drug market share interest in developing a synthetic version of phytocannabinoids. However, in contrast with the properties of phytocannabinoids, which are partial agonists of cannabinoids receptors, synthetic cannabinoids are usually full agonists/antagonists of one receptor. This makes synthetic cannabinoids very useful in scientific research, but very hazardous for public use, because of the increased toxicity [75].

There are different classifications of synthetic cannabinoids. Excluding the colloquial or code names, some compounds are named according to their discovery [76]: the prefix WIN-, followed by a number, was used after the company Sterling-Winthrop discovery of WIN-55,212-2, which effects resemble THC’s ones and indeed it binds to CB1 and CB2 as agonist [77]; the prefix HU-, followed by a number, refer to the discovery of HU-210, which have a similar structure of THC, at Hebrew University of Jerusalem and so, as WIN-55,212-5, exert its action on CB1 and CB2 [78], while HU-308, a CBD-derivative, show a strong selectivity for CB2; the prefix CP-, followed by a number, refers to a group of compounds synthetized by Pzifer Inc. in the decade 1970–1980 [76]; JWH-: the prefix JWH-, followed by a number, refer to the work of Jhon W. Huffman’s research group at Clemson University in North Carolina. JWH-018 is considered the base of aminoalkylindole, and gives rise to a large variety of similar synthetic cannabinoids [79] as JWH-133, selective for CB2; the prefix AM-, followed by a number, refer to the work of Alexandros Makiryannis’s group, which synthetized a wide variety of synthetic cannabinoids with different structures and receptors selectivity [76]. As example of the variety of AM- compounds and their different affinity, AM-1241 is selective for CB2, while AM-404 has agonist action on CB1 and TRPV1. The compounds whose effects will be mentioned in this review are listed in Table 1.

The European Monitoring Center for Drugs and Drugs Addiction (EMCDDA) has classified synthetic cannabinoids according to their structure [80]. Their chemical structure can be divided into four main groups: core, tail, linker and linked.

The core makes the difference regarding the receptor affinity. It has been proved that indazole and indole have a better affinity to CB1, while carbazole group or, in general, its larger group have increased affinity with CB2 [76,81]. The length of the tail group, a small portion of the molecule attached to the core group, can modify the affinity of the compound to the receptors, in particular CB1. It seems also that this affinity is mainly modified at CB1 level [82]. The linker group forms a bridge between core and linked groups. It can be carbonyl, ester or amide, with different combinations of cores and linked group [83]. The last group, the linked one, can be divided in other two sub-groups, the cyclic-linked and the non-cyclic linked. The size of the cyclic group determines the affinity to the receptor, while the pharmacokinetic of non-cyclic linked group is under investigation, especially after the public alert against non-cyclic-linked cannabinoids for their toxicity [76].

**Figure 3:**
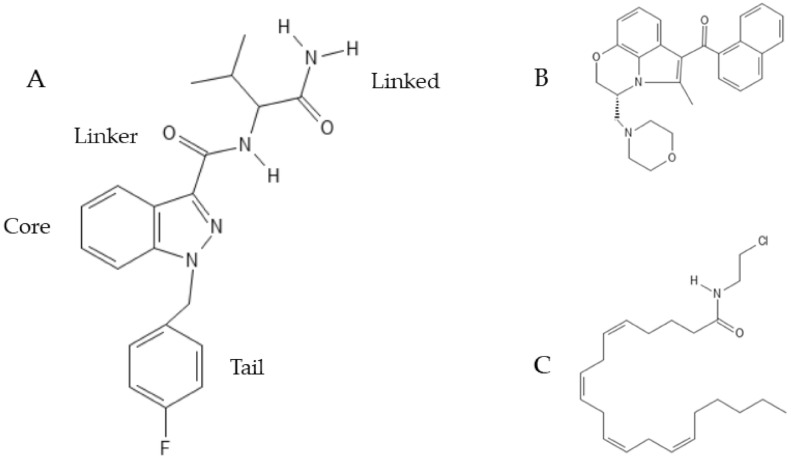
Synthetic cannabinoid structure and chemical structures of two synthetic cannabinoids mentioned in this review. (**A**) General structure of a synthetic cannabinoid according to the European Monitoring Center for Drugs and Drugs Addiction. The different structure of the four groups influences the properties of the drug. (**B**) Chemical structure of WIN-55,212-2; (**C**) chemical structure of Arachidonyl-2’-chloroethylamide (ACEA).

The general structure with the four main groups is reported in Figure 3, along with the structure of two synthetic cannabinoids most mentioned in this review.

## 6. Psychoactive and Non-Psychoactive Cannabinoids Effects on Neurogenesis

### 6.1. Proof-of-Concept Experiments

Before starting the tests of phyto- or synthetic cannabinoids on models of different diseases, it is important to understand influence of cannabinoids in cells or animals that do not resemble any pathological condition. The role of CB1 and CB2 receptors in neurogenesis was evaluated using the knock-out models in different experiments.

The role of CB1 and CB2 in neurogenesis modulation was evaluated in an experiment with cells from SVZ and DG harvested from rats and exposed to different synthetic cannabinoids: ACEA, HU-308 and WIN55,212-2, all at doses of 100 nM, 300 Nm or 1 γM. The results indicate an important difference in neurogenesis depending on the type of receptor-stimulated: the activation of CB1 increases the proliferation in SVZ cells, but the co-activation does not have the same result; the simultaneous activation of both receptors was indeed necessary for cells proliferation in DG. Regarding neuronal differentiation, the selective or simultaneous activation of the receptor seems to all increase the differentiation [84]. Expression of CB1 on NSCs/NPCs resulted to be indispensable for the neurogenesis in DG since its knock-out was able to reduce their proliferation and the total number of new neurons in the DG. It is important to mention that the influence of CB1 in proliferation was specific to DG and was not replicated in SVZ, suggesting that different neurogenic niches have also different regulator mechanisms. 2-AG is also necessary for neurogenesis, but a functional 2-AG is not enough to compensate for the lack of CB1 and restore a proper neurogenesis in DG. This reduction in proliferation also resulted in a reduced number of astrocytes, neuroblasts and mature granule cells. Electrophysiology tests revealed a decrease in LTP in hippocampus, probably due to a reduction in dendritic length and spine density, suggesting the role of CB1 in proper dendritic growth in newborn neurons. It was also enlightens a reduction in BDNF levels in CB1-knockout mice, probably due to an impairment of the signal cascade of CB1, which involves PI3K/AKT and ERK, followed by mTORC1 and cAMP response element-binding protein (CREB) which regulates the expression of genes such as *BDNF*, but also *FOS* and *JUN* which play an important role in cell survival [85]. Surprisingly, the lack of CB2 does not seem to have any effect on the neurogenesis nor the ratio of cell proliferation in unstimulated brain, which lead to thinking that its role may be exert only in specific conditions, particularly, when neurogenesis needs to be dynamically regulated [86].

As mention before, BDNF and neurogenesis appear to correlate, not only because BDNF is expressed after cannabinoid stimulation. Different synthetic cannabinoids were used to evaluate the crosstalk between endocannabinoid receptors and BDNF (30 ng/mL), and they were ACEA, HU-308 and WIN-55212-2, all at dose 1 μM, along with selective CBs antagonists. Results showed that CB2 influences the effect of BDNF in DG, while CB1 and CB2 exert a modulation on the BDNF-neurogenesis in SVZ. BDNF seems to be a necessary requirement for cannabinoid action in both DG and SVZ, suggesting regulation of neurogenesis by crosstalk between cannabinoid receptors and BDNF. To confirm the previous mention evidence that the two neurogenic niches have different mechanisms involved in neurogenesis regulation, the selective antagonism of the CBs receptor seems to have an effect on BDNF neurogenesis restricted in DG and to the early stages. Regarding the cell proliferation profile, CB1 plays a role in SVZ, while the co-activation of CB1 and CB2 seems required in DG. Both receptors activation can induce neuronal differentiation. The link between BDNF and neurogenesis might be CREB protein because cannabinoids showed to stimulate its phosphorylation and increase both BDNF and CREB expression, and CREB itself is a regulator of BDNF expression [87]. Additionally, CREB proved to play a role in neurogenesis, since experiments with an inhibitor of cyclic adenosine monophosphate (cAMP) scavenger increased proliferation of newborn hippocampal cells, and it can also have an effect of differentiation since it was located in immature neurons [88]. A confirmation of CREB role in differentiation came from evidence where its selective deactivation impaired neuron maturation in SVZ and, in DG, the branching and cellular processes of the granule cells were decreased [89].

An experiment with mice embryonic stem cells after neural induction evaluated the effect of THC in neuronal differentiation. The results suggested that THC (2 μM) can induce neuronal differentiation of the neurons of the deep layer, and this occurs concomitants with the reduction of upper layer neurons transcript, indicating that deep layer neurons differentiation occurs at the expense of the upper layer ones. CB1 signal, in presence of THC, promotes the activity of the transcription factor B-cell lymphoma/leukemia 11B (*BCL11B*), which in turn generate neurons expressing transmembrane protein deleted in colorectal cancer (Dcc) and reduced level of UNC-5 netrin receptor C (*Unc5C)* [90]. *BCL11B* seems to be necessary in order to properly differentiate the cells in the DG because its decreased expression resulted in abnormal cytoskeletal development and a non-functional architecture in granule layer. Moreover, its knockdown in hippocampus provokes impairment in spatial learning and memory tasks, as a result of an alteration in the mossy fiber projection structure. More in general, *BCL11B* regulates progenitors proliferation, apoptosis of post-mitotic neurons and differentiation of cells in DG, but it is also necessary for neurons to be fully integrated into the hippocampal circuit. It has been suggested that *BCL11B* could exert its effects on neurogenesis through interaction with cyclin-dependent kinase inhibitor family members, in particular, it represses the activity of a protein encoded by cyclin-dependent kinase inhibitor 1C *(CDKN1C)* gene, p57KIP2. It regulates the cell cycle, indeed is expressed in differentiated neurons, where the progression of cell cycle is no longer necessary and is repressed in the area where differentiation is active [91]. The regulation of differentiation from *BCL11B* is again mediated by ERK and AKT but was found to be independent by cAMP, mTORC1 and JNK, suggesting that the activation of a receptor does not necessarily provoke the same signal cascade in different areas or different types of neurons. Patch-clamp analyses indicated neurons that originated under THC stimulus are fully functional. However, THC-exposed human induced pluripotent stem (hiPS) derived neurons have altered expression of genes involved in neurodevelopment and synaptic function, resemble the autism disorder spectrum, as the altered balance between deep and upper layer development is typical of neuropsychiatric developmental diseases [90].

In line with the previous evidence, low doses of THC can induce effective neurogenesis in the hippocampus of rats, which resulted also in an improvement in cognitive tasks. The effect of THC (0.75, 1.5 and 3 mg/kg) seems to be present at different stages of neurogenesis: the upregulation of nestin suggests an increase in proliferation, while an increase of DCX indicates a successful migration and differentiation, this latter supported by the levels of Tuj-1, which indicates, also, neuron survival. Focusing on DCX, it is important to mention that this marker also increases after exercise or after putting the animal in an enriched environment, so DCX level is positively associated with neurogenesis and can explain the improvement of cognitive tasks. As mentioned in a previous study, BDNF level was found to increase and, along with DCX levels, demonstrated an efficient synaptic plasticity [92].

The second phytocannabinoid, CBD, also proved to have effects on neurogenesis, but it is dose-dependent. Repeated doses of CBD (3, 10 and 30 mg/kg) have a comparable effect as imipramine in reducing anxious behaviors, and lower dose of CBD increase cell proliferation and neurogenesis. On the contrary, chronic high doses of CBD show opposite effects on proliferation and neurogenesis, maintaining the antidepressant properties similar to the benchmark. This apparently weird behavior of CBD can be explained by the desensitization of CB1 receptor, which after high repeated doses of CBD is no longer able to promote neurogenesis, resulting in a decreased number of DCX-positive cells [93].

Wondering which phytocompound can promote better neurogenesis, a comparison was made between THC and CBD administration in mice. Chronic administration of THC (41.2%) seems to not have any effect on neurogenesis, while it impairs cognitive functions and the mice performed better in the Rotarod tests, even if other evidence points to a decreased of locomotor activity after THC administration. Mice fed with CBD (38.8%) had the same score as controls in Morris Water Maze, even if it enhances neurogenesis. The neurogenic effect of the CBD was blocked in the CB1^-/-^ mice, even if there was increased proliferation, suggesting that CBD effect is due to CB1 signaling [94].

Phytocompounds are not full agonists of CBs receptors, so different tests evaluated, also, the effect of synthetic cannabinoids in neurogenesis. HU-210 (25 and 100 ng/kg) is able to promote the proliferation of embryonic NSCs and NPCs using CB1, and in particular, ERK signaling seems to be activated. Even if neither HU-210 nor AEA seems to induce differentiation, the increased proliferating ratio of NSCs and NPCs resulted in an augmentation of newborn hippocampal neurons. It is important to mention that the chronic administration of HU-210 did not lead to neuronal death in hippocampus, so the newborn neurons are resulting from an active induction and not from a compensatory mechanism. In addition to neurogenesis effect, HU-210 proved its anxiolytic and antidepressant effect in rats. The antidepressant effect was measured with a reduced immobility in force swimming test (FST). To explain different results obtained from another research group, the authors underscore the characteristic of cannabinoids to exert different effects according to their dose and frequency of administration, such as acute and high dose of CD1 agonist produce anxiety effects on rats, while chronic and high dose of HU-210 has the opposite effect [95]. Moreover, ACEA (10 mg/kg) and PMSF (30 mg/kg) combination, a protector of ACEA degradation, significantly increase the number of proliferating cells, as well as the combination of the three drugs. The increase of NeuN marker also indicates a positive effect on neurogenesis exert by ACEA+PMSF [96].

Considering O-1602 (4 μg/kg per day) important feature to be an agonist of GPR55 receptor, the contribution of this receptor to neurogenesis can be investigated since both murine and human NSCs express GPR55 in a comparable amount as CB1 and CB2, but its levels decreased during differentiation of human NSCs. Treatment with endogenous and synthetic agonists of GRP55 increases human NSCs proliferation. Continuous infusion of O-1602 into the hippocampus increased cells proliferation and the number of immature neurons, that survived more than in the control group. GPR55^-/-^ mice showed fewer cells proliferation in SGZ compared to the wt mice, and the treatment with O-1602 does not have any effect on cells proliferation in GPR55^-/-^ mice. The activation of GPR55 promotes neuronal differentiation. Similar to the other CBs receptors, GPR55 signaling also involves ERK1/2 phosphorylation and nuclear translocation of NF-κB after CREB activation [97].

An evaluation focusing on the antagonism of TRPV1 has a positive effect on neurogenesis showed that WIN-55212-2 (2 mg/kg per day) can decrease microglia activation in CA3 and DG and antagonizing TRPV1 can have the same effect in DG, reducing also the entry of the calcium ions in the neurons. WIN-55212-2 exert its anti-inflammatory function also in reducing the level of pro-inflammatory cytokines, such as tumor necrosis factor alpha (TNFα), interleukin 1-beta (IL-1β) and interleukin 6 (IL-6). On other hand, there is an increased of interleukin-1 receptor antagonist (IL1-RA), whose function is to act as an anti-inflammatory cytokine. WIN-55212-2 can have a positive effect on neurogenesis only if both CBs receptors are present since the blocking of at least one of them is sufficient to prevent the expected increase in neurogenesis due to WIN-55212-2 treatment [98].

Inflammation is thought to be one of the responsible causes of neurodegeneration, so O-1602 (4 μg/kg per day) capacity of induce neurogenesis in inflammatory conditions was tested. It was found that also systemic inflammation, such as the one resulting from LPS injection, can reduce the neurogenesis through the impairment of NSCs survival and born of new immature neurons, even if the proliferation of NSCs in SVZ remains unaltered. The administration of O-1602 significantly increased the percentage of cells expressing the neuronal marker. The treatment with O-1602 in vitro increased the proliferation of hippocampal NSCs and the number of immature neurons, with no effect on microglia activation. In vivo, it has neuroprotective effects [99].

**Figure 4:**
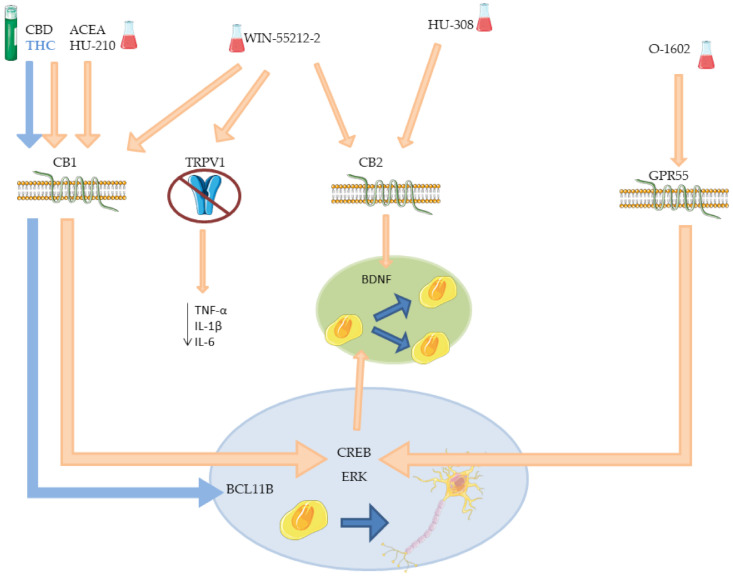
Mechanism of different phyto- and synthetic cannabinoids on neurogenesis. The picture offers a schematic representation of the receptors involved in neurogenesis and their signal cascade activated after agonist action of phyto- or synthetic cannabinoids, tested on cells or animals which do not resemble any specific disease. THC effect on *BCL11B* is highlighted in blue. BCL11B: B-cell lymphoma/leukemia 11B; BDNF: brain-derived neurotrophic factor; CB1: cannabinoid receptor 1; CB2: cannabinoid receptor 2; CBD: cannabidiol; CREB: cAMP response element-binding protein; ERK: extracellular signal-regulated kinases; GRP55: G protein-coupled receptor 55; IL-1β: interleukin 1 beta; IL-6: interleukin 6; THC: Δ^9^-tetrahydrocannabinol; TRPV1: transient receptor potential cation channel subfamily V member 1.

The results discussed so far are resumed in Table 2 and their molecular interactions are schematized in Figure 4.

### 6.2. Cannabinoids Effects on Neurogenesis in AD, PD, HD and HIV-Associated Dementia

AD is the main form of dementia, characterized by neuronal loss, which induces cognitive impairment, and by the presence of Aβ plaques and neurofibrillary tangles of Tau protein [100]. Dementia is also part of the symptoms of PD and Huntington’s disease, however, motor impairment is indeed the most evident symptom [101,102].

The neurotoxicity of Aβ is thought to be one of the most important causes of neuronal death. Neuronal culture exposed to Aβ reacts with a shortening in neurite length, but this can be reversed after treatment with CBD (20 mg/kg) and the effect is CB1-dependent., Moreover, CBD protects AEA from deactivation, reducing the amount of fatty acid amide hydrolase (FAAH), the enzyme responsible for AEA degradation. The same protective mechanism is suggested as explanation of neurogenesis rescue also in genetic AD mouse models [103,104]. In this way, an increased amount of AEA can interact with CB1 and its downstream signal, involving the PI3K/AKT pathway and signal transducer and activator of transcription 3 (STAT3) subsequently, mediate neurite outgrowth. The cognitive dysfunctions, in particular memory loss, in AD are mainly due to a massive loss of hippocampal neurons, indeed Aβ can decrease the amount of synaptic proteins, and altered the dendritic spine density in hippocampus. Treatment with CBD increased the expression of synaptophysin and synapsin I, both expressed on synapsis, indicating a role in inducing synapsis formation or in potentiating the already existent ones. Furthermore, the activation of tropomyosin receptor kinase A (TrkA) receptors was found [104]. TrkA is the main target of nerve growth factor (NGF) and both seem to regulate also apoptosis in neurons. In another experiment, CBD (1 μM) decreased the level of caspase-3 activating TrkA and failed to induce neuritogenesis in cells lacking TrkA [105].

CBD (10 mg/kg) is also capable of promote neurogenesis in rats after injection of Aβ and decreasing neuroinflammation. The activation of microglia and the secretion of pro-inflammatory molecules, caused by Aβ deposition, is one of the causes of neuronal loss in AD patients in a self-sustaining mechanism, where inflammation induces neuronal death, which, in turn, activates more microglial cell and consequently, pro-inflammatory signals. In this scenario the anti-inflammatory properties of CBD can be useful for preventing neuronal death due to excessive microglia activation. Following CBD administration reduce GFAP protein expression, with consequent gliosis impairment, and decrease, also, the release of pro-inflammatory cytokines in astrocytes. Additionally, in vivo, closed to Aβ deposition area, was possible to observe a reduction in astrocytes activation and, also, the rescue of neuronal viability after CBD treatment. These effects seem all to be induced by PPARγ activation since the administration of its antagonist completely abrogate them. The neurogenesis was taking place in DG of hippocampus, in the granule cells layer and this is in opposition to Aβ-induced neuronal loss [106] and probably this effect is correlated with ERK activation, since a strong ERK activation was found after PPARγ agonist administration, even if it has to be pointed out that the dose was moderate. This is important because high doses have the opposite effect, so an excessive stimulation of PPARγ results in ERK inhibition. PPARγ stimulation can also increase the level of cyclin B in NSCs treated with PPARγ agonist, and this is associated with proliferation. However, it has to be pointed out that PPARγ activation is correlated with the impairment of NSCs differentiation, since the phosphorylation only of STAT3 among the STAT family, was found in embryonic mouse stem cells in undifferentiated state [107].

Activation of PPARγ seems also to be responsible for the neurogenesis in the Huntington’s disease model, where VCE-003.2 promoted neural differentiation of murine embryonic stem cells and the number of cells in SVZ. Designed as an orphan drug for Huntington’s disease, VCE-003.2 (10 μg/kg) administration proved to influence neurogenesis in SVZ at different stage: NeuN marker suggests effective neurogenesis followed by neuronal differentiation, as DCX expression indicate; GFAP and KI-67, along with achaete-scute homolog 1 (Ascl1), indicate the mobilization of NSCs [108].This seems in contrast with what was mention before, but other experiments elucidate that the activation of PPARγ at a ground state can induce neuronal differentiation, while its high activation seems to not change the neurogenesis ratio [109]. Probably the dose of cannabinoid administrated could make the difference between the different effects of PPARγ activation on neurogenesis. Since VCE-003.2 is an analog of CBD, its action on PPARγ is quite similar, because it was found to induce the activation of ERK and mTORC1 pathway, in the first moments after administration, while AKT was activated to a higher extent and for a longer time [110].

AM-1241 (0.75, 1.5, 3 and 6 mg/kg) was able to regenerate dopaminergic neurons in a mouse model of PD. Mice were injected with 1-methyl-4-phenyl-1,2,3,6-tetrahydropyridine (MPTP), which can mimic PD’s symptoms. In treated mice, AM-1241 was found a co-localization of CB2 and tyrosine hydrolyse (TH), a marker of dopaminergic neurons, suggesting that the action of AM-1241 on regeneration involves CB2. Moreover, the expression of CB2, Parkin/PTEN-induced kinase 1 (PINK1) and PI3K/AKT in substantia nigra and hippocampus was restored. Probably, as for the previous evidence, P13K/AKT pathway is responsible for the regrowth of dopaminergic neurons, while Parkin/PINK1 plays a role in neuroprotection [111]. We had also shown that CBD, along with cannabigerol (CBG) both at doses of 1 or 5 μM, is able to upregulate the expression of dopaminergic receptors 2 and 4 in NSC-34 cells, inhibiting at the same time the expression of monoamine transporter 2, decreasing in such way dopamine uptake [112], and this may also have a role in synaptic plasticity.

Similar results were also obtained in a mouse model of neurodegeneration caused by HIV, whose glycoprotein Gp120 is involved in HIV-associated dementia and can inhibit hippocampal neurogenesis. Moreover, Gp120 is neurotoxic and can induce neuroinflammation via microglia activation. The consequent neuronal loss leads to cognitive impairment and other dementia-like symptoms. In hNPCs, Gp120 blocked proliferation and induced their apoptosis, while ACEA was able to prevent these damages on hNPCs. In vivo, AM-1241 (10 mg/kg) treatment increased the number of DCX positive cells, indicating the capacity to induce neuroblasts and neuronal proliferation, probably via mTORC signaling, while it impairs, at the same time, astrogliosis and gliogenesis, reducing the inflammation [113]. It has been shown that CB2 and DAGLs are connected in SVG neurogenesis, meaning that they are required for efficient proliferation of neuronal progenitors [114], and endocannabinoid signaling promotes axonal growth, as well as DAGL-dependent CBs signaling; this signaling is downstream of cell adhesion molecules (CAM)/fibroblast growth factor receptors (FGFR) signaling and this allows the access of calcium into the axonal growth cone. Calcium influx is indeed required for axonal growth response [115,116].

### 6.3. Cannabinoids Effects on Neurogenesis in Stroke and Hypoxia/Ischemia Models

Neurodegeneration and cognitive or motor impairment can be due to a disease such as AD or PD, but one must not forget how accidents or traumas can provoke damages in the brain: ischemia and hemorrhagic stroke are just examples of vascular accidents which can have neuronal loss as one of the outcomes.

CBD (10 mg/kg) proved to improve the cognitive performance of mice after brain ischemia. Indeed, cognitive impairment is part of the consequences of brain ischemia, and this could also have impacts on emotions. For this reason, also the effect of CBD in anxiety-like behavior was evaluated. Along with emotional modifications, also the loss of hippocampal cells was noticed after the induction of the model, and the rise of neuroinflammatory markers suggests that different mechanisms act in concert to increase the loss of neurons. This could be responsible for the memory loss, evident in behavioral tests score. Hippocampal neurogenesis is a spontaneous event that contribute to the repair of brain-damaged areas, and it was induced after short-term CBD treatment, as the increased DCX positive neurons indicated. Moreover, the augment of BDNF level in hippocampus after treatment could have given a contribution to neurogenesis induction. It was proposed that CBD can also prevent dendritic degeneration and induce their regeneration by increasing the level of microtubule-associated protein 2 (MAP-2), a microtubule protein that plays a role in synaptic formation. Due to the ability of CBD to activate different receptors, and the lack of tests using selective antagonists, it is unclear which one is responsible for the increased neurogenesis seen in this model [117].

The synthetic WIN-55212-2 (1 or 9 mg/kg) seems to exert its effects in ameliorating hypoxia-ischemia outcome through increased oligodendrocyte progenitors proliferation, probably using ERK pathway. It was noted that, after induction of the model, damaged oligodendrocytes expressed Tau in its dephosphorylated form, while the WIN-55212-2 treated group showed fewer cells positive for dephosphorylated Tau. Dephosphorylation of Tau is known to correlate with apoptosis and its increment happens usually after an insult, such as oxidative stress. Indeed, the cells positive for dephosphorylated Tau were found close to the penumbra area. Higher levels of CB1 were found few hours after the induction of the model and the use of a selective antagonist proved that WIN-55212-2 exerts part of its effect using CB1 signaling pathway, even though a partial contribution of CB2 cannot be excluded. After treatment, in the penumbra area, there was an increase of Ki67/BdrU positive cells in the NG2 positive population, indicating an efficient proliferation of oligodendrocytes progenitor population. It is worth mentioning that, in the non-treated group, there was also verified an increase in cell population, but it did not express NG2, indicating a different type of cells in active spontaneous proliferation [118]. The aim of the oligodendrocytes is to wrap the axons in myelin, in order to speed up the communication between neurons using saltatory conduction. WIN-55212-5 (9 mg/kg) treatment can have a positive effect on the re-myelination after stroke and this happens at different stages of the oligodendrocytes maturation. Oligodendrocytes progenitors cells can be induced into differentiation by WIN-55212-2 through regulation of ERK1/2 phosphorylation, and effective differentiation was proved by the increased number of NG2 positive cells in the peri-infarct area. The level of phosphorylated ERK1/2 was found increased in the treated group, an event not replicated after selective antagonism of CB1, suggesting that CB1 signal cascade is involved in oligodendrocytes differentiation through ERK1/2 phosphorylation. It is important to mention that the over expression of the phophoERK is hazardous for the survival of the cells, since it increased apoptotic markers such as cleaved-caspase3. This suggests that the beneficial effect of WIN-55212-2 may be only partially due to this signal cascade [119]. Oligodendrocytes can synthetize 2-AG, which plays a role in oligodendrocytes progenitors differentiation, probably using the ERK/MAPK pathways such as NPCs: the alternate blocking of CBs decreased phosphorylation of ERK and this effect can be reversed by 2-AG. In light of previous evidence where the impaired ERK activation resulted in impaired oligodendrocytes progenitors differentiation, is correct to think that oligodendrocytes progenitors differentiation occurs in a 2-AG/ERK dependent pathway [120].

WIN-55212-2 (1 mg/kg) also increased the number of oligodendrocytes and neuroblasts in a model of neonatal hypoxia/ischemia. In order to evaluate if the oligodendrocytes examined were newly formed, BrdU staining was performed and it was clear an increase both in BrdU and in BrdU negative NG2 expressing cells; a possible explanation to this phenomenon is that WIN-55212-2 can increase the proliferation of oligodendrocytes progenitors, but also it can promote their migration. WIN-55212-2 treatment-induced can also increase in CB2 expression in SVZ, along with increased cell proliferation in that area, pointing at SVZ as area for newly generated oligodendrocytes progenitors. Neuroblasts number was also found to be increased after WIN-55212-2 treatment, as the increased of DCX marker indicate, and they were located in the injured striatum, however their differentiation in mature neuron was limited, probably due to the shortage period of treatment. However, it is known that CB1 is upregulated too during neurogenesis following brain injury, so it cannot be excluded that a prolonged administration of WIN-55212-2 would have some effects on the differentiation of neuroblasts into neurons [121].

CB2 seems also to be responsible of neurogenesis after stroke, as the administration of JWH-133 (1 μM/L) elucidated in stroke mouse model. The synthetic cannabinoid, through activation of CB2, promotes neuroblasts migration and this may be due to enhanced proliferation. The evidence suggests an active migration comes from the observation of a high amount of neuroblasts in corpus callosum of treated animals, in contrast with no variation in number of proliferating cells in SVZ. On the contrary, selective antagonism of CB2 decrease the amount of DCX positive cells in corpus callosum and increased the ones in SVZ, indicating an important role of CB2 in cell migration. The results were replicated in vitro, where JWH-133 was able to induce NPCs migration [122]. CB2, like CB1, can regulate the expression of genes known to encode for molecules that regulate migration, such as myosin 1C or cell division cycle 42 (CDC42). Moreover, 2-AG seems to play a role during neuroblasts migration, since its abrogation impairs the migration, and its synthesis enzyme is expressed on migrating neuroblasts [123].

### 6.4. Cannabinoids Effects on Neurogenesis in Acute and Chronic Stressed Animals

The effect of CBD on stressed animals was evaluated, and CBD revealed capacity to relieve the anxiety. After exposure to stress stimuli, the decreased of hippocampal neurogenesis was confirmed by the reduction of DCX/BrdU positive cells. CBD (30 mg/kg) administration was able to reverse this process, and the effect of the phytocannabinoid in neuron differentiation was confirmed with an increased level of NeuN, which is a marker of mature neurons. Another important evidence in this experimental set come from the observation that CBD does not modify the behavior of non-stressed mice, but affect the stressed one only if hippocampal neurogenesis was taking place: the hippocampal neurogenesis seems important for the anxiolytic effect of CBD since its abrogation prevent the CBD anxiolytic effects. CBD proliferative action is dose-dependent, and treatment with CBs agonist increased, even more, the cells proliferation. However, it has to be remembered that high doses of CBD can activate also TRPV1, losing the anxiolytic properties of CBD. Administration of CB1 antagonist can block both the anxiolytic and the proliferating effect of CBD, revealing that this function might be CB1-dependent. Moreover, CBD-treated mice showed an increased amount of AEA levels in the hippocampus, since CBD interferes with FAAH-mediated AEA degradation [124].

In chronic stressed animals, CBD (30 mg/kg) exhibits a positive effect on neurogenesis when mediated by CB2, while the concomitant action of CB1 and CB2 contributes to the anxiolytic effect, as well as to the prevention of dendritic remodeling caused by the stress stimuli. Selective antagonists treatment on stressed animals elucidate that CB1 signals have a role in proliferation and survival of the cell not from neuronal origins, while CB2 is more involved in the fate of neuronal progenitors, like their differentiation. CB2 signaling is indeed involved in phosphorylation of glycogen synthase kinase 3 beta (GSK3β), whose hyperactivation resulted in an impairment of NPCs proliferation, and this is probably due to MAPKs activity following CB2 stimulation [125].

The interference in cannabinoids uptake is the base of AM-404 (2 mg/kg) mechanism of action, which effect was tested on stressed rats. When this compound was administrated before the exposure to the stress stimuli, it was able to prevent the appearance of defensive response, and, also, the block of hippocampal cell proliferation. AM-404 is able to act both on CB1 and TRPV1, but usually TRPV1 activation leads to a worse response to stress. The cell proliferation reduction in hippocampus is due to the stress condition, but AM-404 failed to induce proliferation when administered in a normal-state condition [126].

Chronically stressed animals exposed to *Cannabis Sativa*’s smoke revealed the smoke itself contained THC, CBD and cannabinol (CBN), which generate from THC. The amount of these compounds in the animal’s urine was analyzed and the quantity of THC was found to be more 30 times than that CBD. As was already demonstrated by other experiments, stress induces anxiety-like behavior and cannabis smoke does not seem able to prevent this, nor to reduce the anxiety derived by the stress. It is important to mention that animals exposed to cannabis smoke express an unusual tendency to self-grooming, a complex behavior whose underlying mechanisms cannot be elucidated with a simple visual observation: aberrant self-grooming is indeed a characteristic of neuropsychiatric models, as well as “simple” anxiety or stress signals. The cell proliferation of neuronal progenitor, blocked by the stress stimuli, seems to not restart after smoke exposure. However, there was a significant reduction in immature neurons in the DG after treatment, their mobility was increased, and a pronounced dendritic alteration was observed [127].

**Figure 5:**
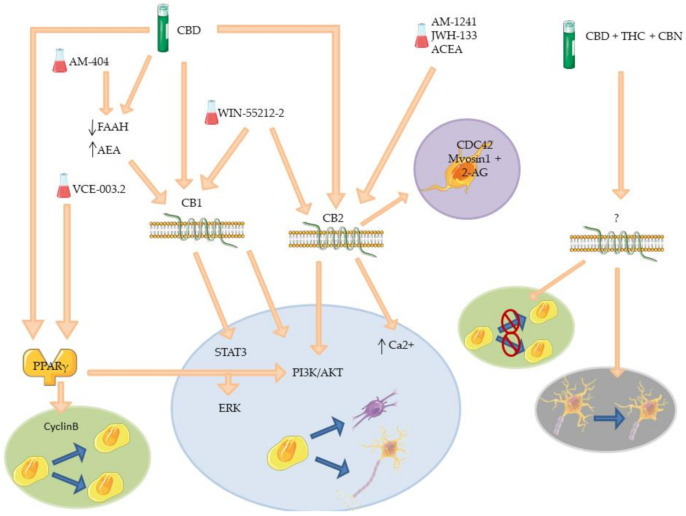
Mechanisms of phyto- and synthetic cannabinoids on neurogenesis in pathological models. The picture offers a schematic representation of the receptors involved in neurogenesis and their signal cascade activated after agonist action of phyto- or synthetic cannabinoids in the mentioned disease models. 2-AG: 2-arachidonoyl glycerol; AEA: arachidonyol ethanolammide or anandamide; CB1: cannabinoid receptor 1; CB2: cannabinoid receptor 2; CBD: cannabidiol; CBN: cannabinol; CDC42: cell division cycle 42; ERK: extracellular signal-regulated kinases; FAAH: fatty acid amide hydrolase; PI3K/AKT: phosphoinositide 3-kinases/protein kinase B; PPARγ: peroxisome proliferator- activated receptor gamma; STAT3: signal transducer and activator of transcription 3; THC: Δ^9^-tetrahydrocannabinol.

Effects of cannabinoids on neurogenesis during the discussed pathological conditions are resumed in Table 3 and the schematization of the effects is reported in Figure 5.

## 7. Conclusions

The current results of cannabinoids effects on neurogenesis are encouraging, and it is expectable that the amount of evidence continues to increase in the future with other experiments. The phytocannabinoid CBD proved its versatility in the treatment, increasing neurogenesis in the models where it was tested. Moreover, it is known to have anxiolytic and anti-inflammatory properties, which can be welcome effects in pathologies such as AD or PD. Regarding synthetic compounds, WIN-55212-2 seems a good candidate for its capacity to activate both CBs receptors, but its effects on pure neurogenesis in pathological conditions are not so prominent. VCE-003.2 and AM-12441 administration proved to have a more direct outcome in neurogenesis. However, CBD exerts its effect, also, on PPARγ, so it is probably the current best candidate for a neurogenesis-based therapy. Further experiments are necessary to evaluate side effects and to standardize the treatment.

## Figures and Tables

**Table 1:** Synthetic cannabinoids. List of synthetic cannabinoids mentioned in this review, with the receptor where they normally exert their effect.

Name	Receptor	Action (Agonist/Antagonist)
ACEA	CB1	Selective agonist
AM-1241	CB2	Selective agonist
HU-210	CB1 or CB2	Non-selective agonist
HU-308	CB2	Selective agonist
JWH-133	CB2	Selective agonist
O-1602	GPR55	Agonist
VCE-003.2	PPARγ	Agonist
WIN-55212-2	CB1 or CB2 TRPV1	Non-selective agonist Antagonist
AM-404	CB1 and TRPV1	Agonist

**Table 2:** Proof-of-concept of cannabinoids capacity to induce neurogenesis, with the receptor(s) where they are supposed to exert the effects described in the selected works.

Compound	Receptor(s)	Effects	References
ACEA	CB1	Increased proliferation in SVZ. Increased neuronal differentiation.	[86,87,96]
HU-308	CB2	Increased proliferation in SVZ and DG. Increased neuronal differentiation.	[86,87]
WIN-55212-2	CB1 and CB2 TRPV1	Increased proliferation in SVZ and DG. Increased neuronal differentiation. Decreased microglia activation. Decreased microglia activation in DG.	[86,87,98]
THC	CB1	Neuronal differentiation in deep layer. Reduce neurons in upper layer. Altered expression of neurodevelopmental and synaptic function genes. Increased neurogenesis in hippocampus. Increased/decreased cognitive performance †. Worsening/ameliorate locomotion †.	[90,92,94]
CBD	CB1	Increased cell proliferation (low dose). Increased neurogenesis (low dose). Reduce anxiety behaviors. Reduced cell proliferation (high dose). Reduce neurogenesis (high dose).	[93,94]
HU-210	CB1	Induced proliferation of embryonic NSCs and NPCs. Increased hippocampal neurons number. Anxiolytic and antidepressant effects.	[95]
O-1602	GPR55	Increased differentiated neurons number. Increase immature neuron number. Increased proliferation in hNSCs. Increased neuronal differentiation.	[97,99]

Some effects in contrast with previous evidences are marked with †. CB1: Cannabinoid 1; CB2: cannabinoid receptor 2; CBD: cannabidiol; DG: dentate gyrus; GRP55: G protein-coupled receptor 55; NPCs: neuronal progenitor cells; NSCs: neural stem cells; SVZ: subventricular zone; THC: Δ^9^-tetrahydrocannabinol; TRPV1: transient receptor potential cation channel subfamily V member 1.

**Table 3:** Cannabinoids and their effect on neurogenesis in pathological models, with the receptor(s) where they are supposed to exert the effects described in the selected works.

Compound	Receptor(s)	Effects	References
CBD	CB1, CB2 and PPARγ	Decreased level of FAAH. Promotion neurite outgrowth. Synapsis formation and protection. Increased neurogenesis. Increase NSCs proliferation. Inhibit NSCs differentiation. Anxiolytic effects. Anti-inflammatory effects.	[103,104,105,106,107,117,124,125]
WIN-55212-2	CB1 and CB2	Re-myelinization. Increased oligodendrocytes progenitors proliferation, survival and differentiation. Increased neuroblast number.	[118,119,121]
ACEA	CB2	Prevention of Gp120-induced hNPCs reduction.	[113]
AM-1241	CB2	Dopaminergic neurons regeneration. Promotion of NSCs differentiation. Anti-inflammatory effects.	[111,113]
JWH-133	CB2	Promotion of neuroblast migration. Promotion of NPCs migration.	[122]
VCE-003.2	PPARγ	Promotion of NSCs differentiation. Promotion of NSCs proliferation.	[108,110]
AM-404	CB1 and TRPV1	Prevention of block of hippocampal cell proliferation. Prevention of defensive behavior.	[126]
CBD+THC+CBN	/	Reduction of immature neurons number in DG. Increased mobility of immature neurons in DG. Dendritic morphology alteration.	[127]

CB1: cannabinoid receptor 1; CB2: cannabinoid receptor 2; CBD: cannabidiol; CBN: cannabinol; DG: dentate gyrus; FAAH: fatty acid amide hydrolase; NPCs: neuronal progenitor cells; NSC: neural stem cells; THC: Δ^9^-tetrahydrocannabinol; TRPV1: transient receptor potential cation channel subfamily V member 1.

## Data Availability

No new data were created or analyzed in this study. Data sharing is not applicable to this article.

## Abbreviations

**Abbreviation**	**Full Text**
2-AG	2-arachidonoyl glycerol
ABHD4	Abhydrolase Domain Containing 4, N-Acyl Phospholipase B
AD	Alzheimer’s Disease
AEA	Arachidonyol ethanolammide or anandamide
ALS	Amyotrophic Lateral Sclerosis
ASCL1	Achaete-scute homolog 1
Aβ	Beta-amyloid
BCL11B	B-cell lymphoma/leukemia 11B
BDNF	Brain-derived neurotrophic factor
BdrU	Bromodeoxyuridine
CAM	Cell adhesion molecules
cAMP	Cyclic adenosine monophosphate
CB1	Cannabinoid receptor 1
CB2	Cannabinoid Receptor 2
CBD	Cannabidiol
CBDA	Cannabidiolic acid
CBG	Cannabigerol
CBGA	Cannabigerolic acid
CBN	Cannabinol
CDC42	Cell Division Cycle 42
CDKN1C	Cyclin-dependent kinase inhibitor 1C
CR	Calretinin
CREB	cAMP response element-binding protein
DAGL	Ciacylglycerol lipase
Dcc	Transmembrane protein deleted in colorectal cancer
DCX	Doublecortin
DG	Dentate gyrus
EMCDDA	European Monitoring Center for Drugs and Drugs Addiction
ERK	Extracellular signal-regulated kinases
FAAH	Fatty acid amide hydrolase
FDA	Food and Drug Administration
FGFR	Fibroblast growth factor receptors
FST	Force swimming test
GABA	Gamma-aminobutyric acid
GDE1	Glycerophosphodiesterase 1
GFAP	Glial fibrillary acidic protein
GPP	Geranylpyrophosphate
GPR55	G protein-coupled receptor 55
GSK3β	glycogen synthase kinase 3 beta
hiPS	Human induced pluripotent stem
IL	Interleukin
IL1-RA	Interleukin-1 receptor antagonist
JNK	c-Jun N-terminal kinase
Ki-67	Antigen Ki-67
KROX-24	Early growth response protein 1
LTP	Long term potentiation
MAP-2	Microtubule-associated protein 2
MAPKs	Mitogen-activated protein kinases
MPTP	1-methyl-4-phenyl-1,2,3,6-tetrahydropyridine
mTORC	Mechanistic target of rapamycin complex
NAE	N-acyl-ethanolamine
NAPE	N-acylphosphatidylethanolamine
NAPE-PDL	N-acylphosphatidylethanolamine phospholipase D
NAT	N-acyltransferase
NeuN	Hexaribonucleotide Binding Protein-3
NeuroD1	Neurogenic differentiation 1
NF-kB	Transcription factors like nuclear factor kappa-light-chain-enhancer of activated B cells
NPCs	Neuronal progenitor cells
NSCs	Neural stem cells
p27Kip1	Cyclin-dependent kinase inhibitor 1B protein
Pals1	Protein associated with Lin-7
PD	Parkinson’s Disease
PI3K/AKT	Phosphoinositide 3-kinases/protein kinase B
PINK1	PTEN-induced kinase 1
PIP2	Phosphatidylinositol 4,5-bisphosphate
PLC	NAPE-phospholipase C
PLCβ	Phospholipase C-β
PPARγ	Peroxisome proliferator- activated receptor gamma
Prox1	Prospero related homeobox gene
PSA-NCAM	Polysialylated-neural cell adhesion molecule
Rhoa	Ras homolog gene family members A
ROCK	Rho-associated protein kinase
SGK1	Serum and glucocorticoid-regulated kinase 1
SGZ	Subgranular zone
SOX2	Sex determining region Y-box 2
STAT3	Signal transducer and activator of transcription 3
SVZ	Subventricular zone of the lateral ventricle
THC	Δ^9^-tetrahydrocannabinol
TLX	Nuclear receptor subfamily 2 group E member 1
TNFα	Tumor necrosis factor alpha
TrkA	Tropomyosin receptor kinase A
TRPV1	Transient receptor potential cation channel subfamily V member 1
Tuj1	β-tubulin isoform III
UNC5C	UNC-5 netrin receptor C
Δ^1^-THCA	Δ^1^-tetrahydrocannabinolic acid
